# Bis{4-[(*E*)-2-(1*H*-indol-3-yl)ethen­yl]-1-methyl­pyridinium} 4-chloro­benzene­sulfonate nitrate

**DOI:** 10.1107/S1600536813030080

**Published:** 2013-11-09

**Authors:** Hoong-Kun Fun, Ching Kheng Quah, Nawong Boonnak, Suchada Chantrapromma

**Affiliations:** a X-ray Crystallography Unit, School of Physics, Universiti Sains Malaysia, 11800 USM, Penang, Malaysia; b Faculty of Traditional Thai Medicine, Prince of Songkla University, Hat-Yai, Songkhla 90112, Thailand; c Department of Chemistry, Faculty of Science, Prince of Songkla University, Hat-Yai, Songkhla 90112, Thailand

## Abstract

In the title mixed salt, 2C_16_H_15_N_2_^+^·C_6_H_4_ClO_3_S^−^·NO_3_^−^, one of the cations shows whole mol­ecule disorder over two sets of sites in a 0.711 (7):0.289 (7) ratio. The 4-chorobenzenesulfon­ate anion is also disordered over two orientations in a 0.503 (6):0.497 (6) ratio. The cations are close to planar, the dihedral angles between the pyridinium and indole rings being 1.48 (3)° in the ordered cation, and 5.62 (3) and 2.45 (3)°, respectively, for the major and minor components of the disordered cation. In the crystal, the cations are stacked in an anti­parallel manner approximately along the *a*-axis direction and linked with the anions *via* N—H⋯O hydrogen bonds and C—H⋯O inter­actions, generating a three-dimensional network. Weak C—H⋯π and π–π inter­actions [with centroid–centroid distances of 3.561 (2)–3.969 (7) Å] are also observed.

## Related literature

For related structures, see: Chantrapromma *et al.* (2008 ▶); Chantrapromma & Fun (2009 ▶). For background to non-linear optical materials, see: Dittrich *et al.* (2003 ▶); Nogi *et al.* (2000 ▶); Raimundo *et al.* (2002 ▶); Ruanwas *et al.* (2010 ▶); Sato *et al.* (1999 ▶).
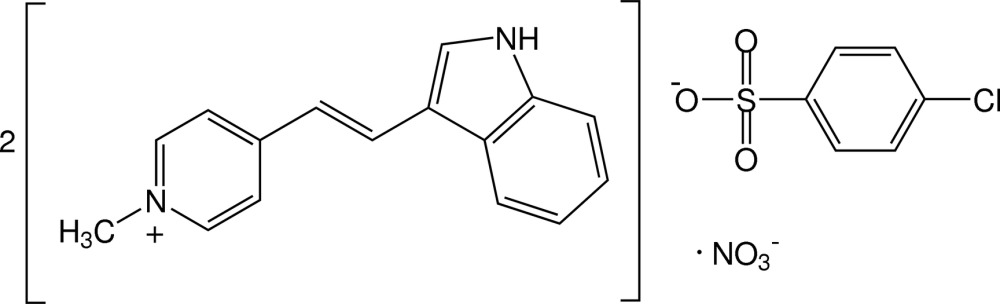


## Experimental

### 

#### Crystal data

2C_16_H_15_N_2_^+^·C_6_H_4_ClO_3_S^−^·NO_3_^−^
*M* *_r_* = 724.21
Triclinic, 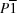

*a* = 8.7540 (7) Å
*b* = 13.6648 (10) Å
*c* = 15.3465 (11) Å
α = 97.206 (1)°
β = 91.186 (2)°
γ = 99.924 (1)°
*V* = 1792.3 (2) Å^3^
*Z* = 2
Mo *K*α radiation
μ = 0.22 mm^−1^
*T* = 153 K
0.55 × 0.47 × 0.14 mm

#### Data collection

Bruker APEXII CCD diffractometer
Absorption correction: multi-scan (*SADABS*; Bruker, 2005 ▶) *T*_min_ = 0.890, *T*_max_ = 0.970
9106 measured reflections
6217 independent reflections
4480 reflections with *I* > 2σ(*I*)
*R*_int_ = 0.021

#### Refinement

*R*[*F* ^2^ > 2σ(*F* ^2^)] = 0.074
*wR*(*F* ^2^) = 0.254
*S* = 1.05
6217 reflections
636 parameters
206 restraints
H-atom parameters constrained
Δρ_max_ = 0.87 e Å^−3^
Δρ_min_ = −0.29 e Å^−3^

### 

Data collection: *APEX2* (Bruker, 2005 ▶); cell refinement: *SAINT* (Bruker, 2005 ▶); data reduction: *SAINT*; program(s) used to solve structure: *SHELXTL* (Sheldrick, 2008 ▶); program(s) used to refine structure: *SHELXTL*; molecular graphics: *SHELXTL*; software used to prepare material for publication: *SHELXTL*, *PLATON* (Spek, 2009 ▶) and *publCIF* (Westrip, 2010 ▶).

## Supplementary Material

Crystal structure: contains datablock(s) global, I. DOI: 10.1107/S1600536813030080/hb7156sup1.cif

Structure factors: contains datablock(s) I. DOI: 10.1107/S1600536813030080/hb7156Isup2.hkl

Supplementary material file. DOI: 10.1107/S1600536813030080/hb7156Isup3.cml

Additional supplementary materials:  crystallographic information; 3D view; checkCIF report

## Figures and Tables

**Table 1:** Hydrogen-bond geometry (Å, °) *Cg*3, *Cg*6, *Cg*7 and *Cg*9 are the centroids of the C16–C21, C32–C37, N4*A*/C30*A*–C32*A*/C37*A* and C32*A*–C37*A* rings, respectively.

*D*—H⋯*A*	*D*—H	H⋯*A*	*D*⋯*A*	*D*—H⋯*A*
N2—H1*N*2⋯O3*A*	0.78	2.19	2.937 (9)	161
N4—H1*N*4⋯O4^i^	0.81	2.43	3.220 (11)	165
N4—H1*N*4⋯O5^i^	0.81	2.32	2.987 (8)	141
C3*A*—H3*AA*⋯O5^ii^	0.93	2.43	3.246 (13)	146
C8—H8*A*⋯O2*A* ^iii^	0.93	2.40	3.213 (8)	146
C10—H10*A*⋯O5^iv^	0.93	2.51	3.234 (6)	134
C18—H18*A*⋯O1*A* ^v^	0.93	2.52	3.345 (8)	148
C22—H22*A*⋯O1*A* ^iii^	0.96	2.45	3.368 (9)	160
C22—H22*C*⋯O2*A* ^vi^	0.96	2.32	3.082 (9)	136
C26—H26*A*⋯O6^vii^	0.93	2.53	3.440 (7)	168
C15—H15*A*⋯*Cg*6^vii^	0.93	2.71	3.550 (6)	151
C15—H15*A*⋯*Cg*7^vii^	0.93	2.94	3.844 (10)	165
C15—H15*A*⋯*Cg*9^vii^	0.93	2.83	3.656 (13)	149
C34—H34*A*⋯*Cg*3^ii^	0.93	2.78	3.602 (7)	149
C38—H38*C*⋯*Cg*6^ii^	0.96	2.95	3.714 (8)	137
C38—H38*C*⋯*Cg*9^vii^	0.96	2.83	3.627 (14)	141
C34*A*—H34*B*⋯*Cg*3^ii^	0.93	2.89	3.56 (2)	130

Symmetry codes: (i) 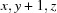; (ii) 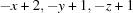; (iii) 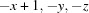; (iv) 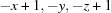; (v) 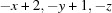; (vi) 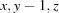; (vii) 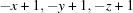.

**Fig. 1.**
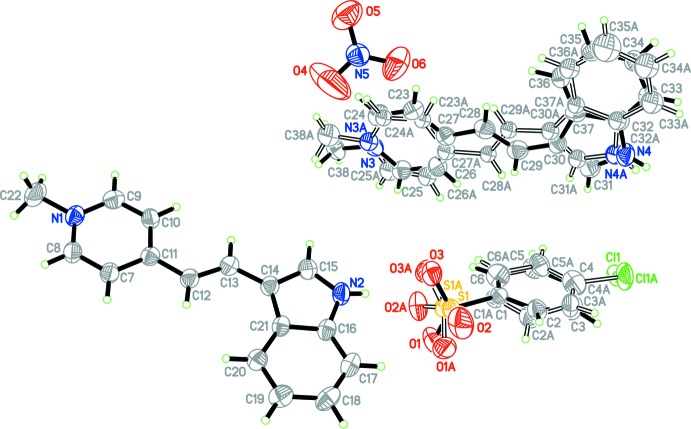
The structure of (I) showing 30% probability displacement ellipsoids. Open bonds show the minor disorder components.

**Fig. 2.**
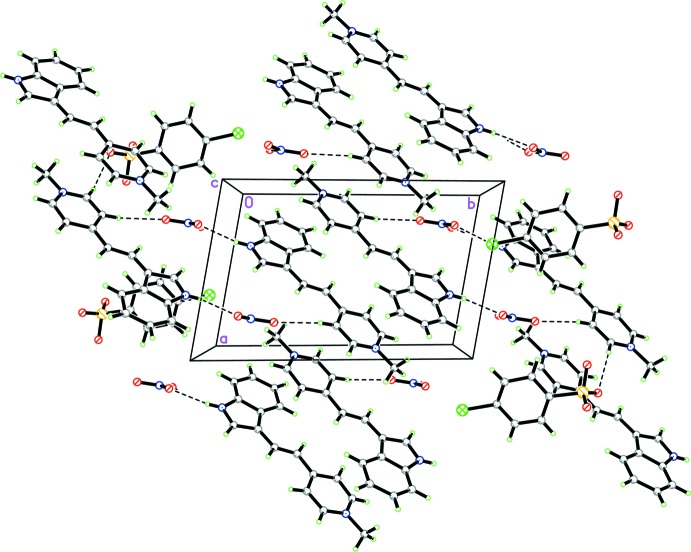
The crystal packing (involving only the major components of the disordered ions) viewed along the c axis. Hydrogen bonds are drawn as dashed lines.

## supplementary crystallographic information

### 1. Comment

Organic molecules that exhibit second-order NLO properties usually consist of a framework with delocalized π system, end-capped with either a donor or acceptor substituent or both. Several pyridinium derivatives have been reported to exhibit second-order NLO properties such as single crystals of 1-methyl-4-(2-(4-(dimethylamino)phenyl)ethynyl)pyridinium *p*-toluenesulfonate (DAST) and its analogues (Dittrich *et al.*, 2003; Sato *et al.*, 1999). Based on the knowledge that the organic dipolar compounds with extended π systems having terminal donor and acceptor groups are likely to exhibit large hyperpolarizability (*β*) (Raimundo *et al.*, 2002), we have synthesized several quinolinium derivatives which exhibit NLO properties (Ruanwas *et al.*, 2010). In a similar manner, the title compound (I) was designed and synthesized in order to study for its NLO property. Unfortunately (I) crystallizes in a centrosymmetric *P*-1 space group which precluded the second-order NLO properties. Herein the crystal structure of (I) is reported.

In the crystal structure of (I), the asymmetric unit consists of two C_16_H_15_N_2_^+^ cations, C_6_H_4_ClO_3_S^-^ and NO_3_^-^ anions (Fig. 1). One cation [C23–C38/N3/N4] exhibits whole molecule disorder over two sets of sites with a refined site-occupancy ratio of 0.711 (7):0.289 (7). The molecule is disordered in such a way that the ethynyl unit in the major and minor (*A*) components are related by a 180° rotation. The two cations exist in the *E* conformation with respect to the ethenyl unit and the torsion angle C11–C12–C13–C14 = -178.8 (3)° for the non-disordered cation, and C27–C28–C29–C30 = 176.8 (6)° and -179.8 (15)° for the major and minor (*A*) components for the disordered cation. The cations are close to planar with the dihedral angles between the pyridinium and the indole rings being 1.28 (3)° for the non-disordered cation, and 5.62 (3) and 2.45 (3)° for the major and minor components respectively for the disordered cation. The 4-chlorobenzenesulfonate anion also shows whole molecule disorder (Fig. 1) with a 0.503 (6):0.497 (6) site occupancy ratio. Bond lengths of the title compound are comparable to those in related structures (Chantrapromma *et al.*, 2008; Chantrapromma & Fun, 2009)

In the crystal (Fig. 2), the cations are stacked in an antiparallel fashion into columns approximately along the *a* axis and are further linked to the anions *via* N—H···O hydrogen bonds and C—H···O interactions (Table 1). C—H···π interactions and π–π interactions were observed with *Cg*1···*Cg*2^iii^ = 3.6804 (19) Å, *Cg*2···*Cg*3^iii^ = 3.561 (2) Å, *Cg*2···*Cg*10^vi^ = 3.969 (7) Å, *Cg*2···*Cg*11^vi^ = 3.949 (7) Å, *Cg*5···*Cg*5^viii^ = 3.729 (5) Å, *Cg*5···*Cg*8^viii^ = 3.728 (8) Å and *Cg*8···*Cg*8^viii^ = 3.741 (11) Å; *Cg*1, *Cg*2, *Cg*3, *Cg*5, *Cg*8, *Cg*10 and *Cg*11 are the centroids of N2/C14–C16/C21, N1/C7–C11, C16–C21, N3/C23–C27, N3A/C23A–C27A, C1A–C6A and C1–C6, respectively [symmetry code (viii) = -*x*, 1 - *y*, 1 - *z*].

### 2. Experimental

4-[(*E*)-2-(1*H*-Indol-3-yl)ethenyl]-1-methylpyridinium iodide (compound A) was synthesized from a mixture (1:1:1 molar ratio) of 1,4-dimethylpyridinium iodide (2.00 g, 8.51 mmol), indole-3-carboxaldehyde (1.24 g, 8.51 mmol) and piperidine (0.84 ml, 8.51 mmol) in methanol (40 ml) under reflux for 2 h under a nitrogen atmosphere. The solid which formed was filtered, washed with ether and recrystallized from methanol to give orange single crystals of compound A after several days. The title compound was synthesized by mixing compound A (0.24 g, 0.67 mmol) in hot methanol (30 ml) and silver(I) 4-chlorobenzenesulfonate (0.20 g, 0.67 mmol) in hot methanol (20 ml). The mixture, which turned yellow and cloudy immediately, yielded a gray solid of silver iodide. After stirring the mixture for *ca.* 30 min, the precipitate of silver iodide was removed and the resulting solution was evaporated to yield an orange solid. Orange blocks of (I) were recrystalized from methanol solution by slow evaporation of the solvent at room temperature after several days.

### 3. Refinement

All H atoms were positioned geometrically and allowed to ride on their parent atoms, with N—H = 0.78, 0.81 and 0.86 Å, CH and C_aryl_—H = 0.93 Å and C_methyl_—H = 0.96 Å. The *U*_iso_ values were constrained to be 1.5*U*_eq_ of the carrier atom for methyl H atoms and 1.2*U*_eq_ for the remaining H atoms. A rotating group model was used for the methyl groups. One cation is whole molecule disordered over two sites with refined site occupancies ratio 0.711 (7):0.289 (7), whereas the 4-chlorobenzenesulfonate anion is disordered over two sites with refined site occupancies ratio 0.503 (6):0.497 (6). Similarity and simulation restraints were applied. The displacement ellipsoids of each of the two pairs of atoms i.e. "CL1 C6" and "N3 C38" were restrained to be almost equal.

### Crystal data

**Table:**

2C_16_H_15_N_2_^+^·C_6_H_4_ClO_3_S^−^·NO_3_^−^	*Z* = 2
*M**_r_* = 724.21	*F*(000) = 756
Triclinic, *P*1	*D*_x_ = 1.342 Mg m^−^^3^
Hall symbol: -P 1	Mo *K*α radiation, λ = 0.71073 Å
*a* = 8.7540 (7) Å	Cell parameters from 6217 reflections
*b* = 13.6648 (10) Å	θ = 2.2–25.0°
*c* = 15.3465 (11) Å	µ = 0.22 mm^−^^1^
α = 97.206 (1)°	*T* = 153 K
β = 91.186 (2)°	Block, orange
γ = 99.924 (1)°	0.55 × 0.47 × 0.14 mm
*V* = 1792.3 (2) Å^3^	

### Data collection

**Table:**

Bruker APEXII CCD diffractometer	6217 independent reflections
Radiation source: sealed tube	4480 reflections with *I* > 2σ(*I*)
Graphite monochromator	*R*_int_ = 0.021
φ and ω scans	θ_max_ = 25.0°, θ_min_ = 2.2°
Absorption correction: multi-scan (*SADABS*; Bruker, 2005)	*h* = −10→10
*T*_min_ = 0.890, *T*_max_ = 0.970	*k* = −11→16
9106 measured reflections	*l* = −17→18

### Refinement

**Table:**

Refinement on *F*^2^	Primary atom site location: structure-invariant direct methods
Least-squares matrix: full	Secondary atom site location: difference Fourier map
*R*[*F*^2^ > 2σ(*F*^2^)] = 0.074	Hydrogen site location: inferred from neighbouring sites
*wR*(*F*^2^) = 0.254	H-atom parameters constrained
*S* = 1.05	*w* = 1/[σ^2^(*F*_o_^2^) + (0.1598*P*)^2^ + 0.6516*P*] where *P* = (*F*_o_^2^ + 2*F*_c_^2^)/3
6217 reflections	(Δ/σ)_max_ = 0.001
636 parameters	Δρ_max_ = 0.87 e Å^−^^3^
206 restraints	Δρ_min_ = −0.29 e Å^−^^3^

### Special details

**Table:**

Geometry. All e.s.d.'s (except the e.s.d. in the dihedral angle between two l.s. planes) are estimated using the full covariance matrix. The cell e.s.d.'s are taken into account individually in the estimation of e.s.d.'s in distances, angles and torsion angles; correlations between e.s.d.'s in cell parameters are only used when they are defined by crystal symmetry. An approximate (isotropic) treatment of cell e.s.d.'s is used for estimating e.s.d.'s involving l.s. planes.
Refinement. Refinement of *F*^2^ against ALL reflections. The weighted *R*-factor *wR* and goodness of fit *S* are based on *F*^2^, conventional *R*-factors *R* are based on *F*, with *F* set to zero for negative *F*^2^. The threshold expression of *F*^2^ > σ(*F*^2^) is used only for calculating *R*-factors(gt) *etc*. and is not relevant to the choice of reflections for refinement. *R*-factors based on *F*^2^ are statistically about twice as large as those based on *F*, and *R*- factors based on ALL data will be even larger.

### Fractional atomic coordinates and isotropic or equivalent isotropic displacement parameters (Å^2^)

**Table:**

	*x*	*y*	*z*	*U*_iso_*/*U*_eq_	Occ. (<1)
O4	0.7782 (12)	0.1160 (6)	0.5522 (4)	0.262 (4)	
N1	0.3365 (3)	−0.3639 (2)	0.08898 (19)	0.0675 (7)	
N2	0.7327 (4)	0.2710 (2)	0.1516 (2)	0.0737 (8)	
H1N2	0.7335	0.3279	0.1670	0.088*	
Cl1	0.6552 (6)	0.9687 (3)	0.3630 (3)	0.0776 (10)	0.497 (6)
S1	0.7770 (6)	0.5393 (3)	0.1964 (3)	0.0744 (15)	0.497 (6)
O1	0.7041 (10)	0.5304 (5)	0.1112 (4)	0.118 (3)	0.497 (6)
O2	0.9408 (8)	0.5430 (5)	0.1947 (6)	0.125 (3)	0.497 (6)
O3	0.7660 (11)	0.4712 (6)	0.2529 (4)	0.085 (3)	0.497 (6)
C1	0.7483 (7)	0.6565 (5)	0.2512 (6)	0.054 (3)	0.497 (6)
C2	0.8661 (9)	0.7372 (6)	0.2595 (12)	0.073 (4)	0.497 (6)
H2A	0.9675	0.7284	0.2493	0.087*	0.497 (6)
C3	0.8328 (13)	0.8344 (6)	0.2838 (18)	0.085 (6)	0.497 (6)
H3A	0.9055	0.8907	0.2763	0.102*	0.497 (6)
C4	0.6954 (12)	0.8448 (4)	0.3177 (9)	0.054 (3)	0.497 (6)
C5	0.5768 (13)	0.7615 (6)	0.3125 (15)	0.073 (5)	0.497 (6)
H5A	0.4798	0.7685	0.3334	0.087*	0.497 (6)
C6	0.6042 (14)	0.6676 (7)	0.2757 (18)	0.104 (9)	0.497 (6)
H6A	0.5235	0.6129	0.2681	0.124*	0.497 (6)
Cl1A	0.7176 (7)	0.9748 (3)	0.3728 (4)	0.1076 (17)	0.503 (6)
S1A	0.7200 (6)	0.5307 (3)	0.1879 (2)	0.0713 (13)	0.503 (6)
O1A	0.8297 (9)	0.5400 (5)	0.1208 (5)	0.115 (3)	0.503 (6)
O2A	0.5651 (8)	0.4921 (4)	0.1546 (5)	0.108 (2)	0.503 (6)
O3A	0.7044 (13)	0.4664 (6)	0.2478 (5)	0.107 (4)	0.503 (6)
C1A	0.7204 (9)	0.6543 (5)	0.2395 (8)	0.063 (3)	0.503 (6)
C2A	0.8482 (11)	0.7269 (6)	0.2379 (12)	0.078 (5)	0.503 (6)
H2AA	0.9299	0.7149	0.2027	0.094*	0.503 (6)
C3A	0.8555 (13)	0.8208 (8)	0.2901 (16)	0.079 (5)	0.503 (6)
H3AA	0.9494	0.8651	0.3001	0.095*	0.503 (6)
C4A	0.7258 (12)	0.8453 (6)	0.3251 (13)	0.108 (8)	0.503 (6)
C5A	0.5943 (14)	0.7713 (7)	0.3256 (16)	0.086 (7)	0.503 (6)
H5AA	0.5067	0.7863	0.3539	0.103*	0.503 (6)
C6A	0.5948 (9)	0.6747 (5)	0.2832 (12)	0.054 (4)	0.503 (6)
H6AA	0.5088	0.6246	0.2851	0.065*	0.503 (6)
C10	0.3905 (4)	−0.1910 (3)	0.1379 (2)	0.0671 (9)	
H10A	0.3681	−0.1366	0.1751	0.080*	
C9	0.3092 (4)	−0.2839 (3)	0.1429 (2)	0.0720 (9)	
H9A	0.2333	−0.2924	0.1842	0.086*	
C8	0.4452 (4)	−0.3513 (3)	0.0303 (2)	0.0693 (9)	
H8A	0.4637	−0.4065	−0.0073	0.083*	
C7	0.5300 (4)	−0.2594 (3)	0.0239 (2)	0.0662 (8)	
H7A	0.6052	−0.2530	−0.0179	0.079*	
C11	0.5065 (4)	−0.1757 (2)	0.0784 (2)	0.0585 (8)	
C12	0.5998 (4)	−0.0784 (2)	0.0709 (2)	0.0616 (8)	
H12A	0.6757	−0.0759	0.0293	0.074*	
C13	0.5851 (4)	0.0068 (2)	0.1188 (2)	0.0644 (8)	
H13A	0.5097	0.0019	0.1606	0.077*	
C14	0.6697 (4)	0.1055 (2)	0.1148 (2)	0.0612 (8)	
C15	0.6401 (5)	0.1873 (3)	0.1677 (2)	0.0735 (10)	
H15A	0.5650	0.1852	0.2097	0.088*	
C16	0.8290 (4)	0.2470 (2)	0.0854 (2)	0.0625 (8)	
C17	0.9405 (4)	0.3088 (3)	0.0471 (3)	0.0734 (10)	
H17A	0.9609	0.3775	0.0649	0.088*	
C18	1.0202 (4)	0.2658 (3)	−0.0180 (3)	0.0835 (12)	
H18A	1.0965	0.3059	−0.0454	0.100*	
C19	0.9891 (4)	0.1619 (3)	−0.0444 (3)	0.0795 (10)	
H19A	1.0464	0.1344	−0.0885	0.095*	
C20	0.8755 (4)	0.0995 (3)	−0.0066 (2)	0.0656 (8)	
H20A	0.8550	0.0309	−0.0249	0.079*	
C21	0.7926 (4)	0.1428 (2)	0.0601 (2)	0.0568 (7)	
C22	0.2475 (6)	−0.4646 (3)	0.0955 (3)	0.1038 (15)	
H22A	0.2297	−0.5021	0.0379	0.156*	
H22B	0.1497	−0.4584	0.1208	0.156*	
H22C	0.3052	−0.4988	0.1320	0.156*	
N3	0.0284 (13)	0.3441 (6)	0.4087 (7)	0.077 (4)	0.711 (8)
N4	0.6488 (10)	0.9026 (5)	0.6160 (5)	0.085 (2)	0.711 (8)
H1N4	0.6724	0.9605	0.6082	0.102*	0.711 (8)
C23	0.2158 (8)	0.3996 (6)	0.5264 (4)	0.080 (2)	0.711 (8)
H23A	0.2707	0.3849	0.5742	0.096*	0.711 (8)
C24	0.1017 (14)	0.3284 (6)	0.4828 (7)	0.086 (4)	0.711 (8)
H24A	0.0737	0.2676	0.5046	0.103*	0.711 (8)
C25	0.0573 (10)	0.4327 (7)	0.3802 (5)	0.0757 (19)	0.711 (8)
H25A	0.0035	0.4431	0.3302	0.091*	0.711 (8)
C26	0.1654 (9)	0.5097 (4)	0.4232 (5)	0.0779 (17)	0.711 (8)
H26A	0.1826	0.5714	0.4021	0.093*	0.711 (8)
C27	0.2506 (6)	0.4966 (4)	0.4985 (4)	0.0668 (14)	0.711 (8)
C28	0.3677 (6)	0.5739 (4)	0.5491 (3)	0.0780 (17)	0.711 (8)
H28A	0.4153	0.5552	0.5976	0.094*	0.711 (8)
C29	0.4126 (6)	0.6668 (4)	0.5332 (3)	0.0790 (17)	0.711 (8)
H29A	0.3676	0.6831	0.4827	0.095*	0.711 (8)
C30	0.5181 (6)	0.7450 (4)	0.5811 (4)	0.0654 (14)	0.711 (8)
C31	0.5477 (9)	0.8421 (7)	0.5594 (4)	0.0832 (19)	0.711 (8)
H31A	0.5021	0.8622	0.5109	0.100*	0.711 (8)
C32	0.693 (2)	0.8500 (7)	0.6800 (10)	0.071 (8)	0.711 (8)
C33	0.7919 (17)	0.8825 (6)	0.7527 (8)	0.071 (3)	0.711 (8)
H33A	0.8419	0.9489	0.7636	0.085*	0.711 (8)
C34	0.8158 (9)	0.8159 (6)	0.8083 (4)	0.0694 (19)	0.711 (8)
H34A	0.8819	0.8379	0.8577	0.083*	0.711 (8)
C35	0.7454 (9)	0.7179 (5)	0.7937 (4)	0.0687 (15)	0.711 (8)
H35A	0.7656	0.6741	0.8324	0.082*	0.711 (8)
C36	0.6398 (9)	0.6815 (4)	0.7187 (5)	0.0673 (16)	0.711 (8)
H36A	0.5898	0.6151	0.7091	0.081*	0.711 (8)
C37	0.6142 (10)	0.7501 (4)	0.6598 (6)	0.062 (2)	0.711 (8)
C38	−0.0857 (7)	0.2599 (6)	0.3651 (5)	0.086 (2)	0.711 (8)
H38A	−0.1657	0.2851	0.3356	0.129*	0.711 (8)
H38B	−0.1309	0.2196	0.4081	0.129*	0.711 (8)
H38C	−0.0352	0.2197	0.3229	0.129*	0.711 (8)
N3A	0.027 (3)	0.3295 (12)	0.4146 (17)	0.068 (8)*	0.289 (8)
N4A	0.634 (2)	0.8785 (9)	0.6083 (10)	0.068 (6)*	0.289 (8)
H2N4	0.6593	0.9378	0.5939	0.082*	0.289 (8)
C23A	0.2220 (17)	0.4290 (9)	0.5133 (9)	0.054 (4)*	0.289 (8)
H23B	0.2833	0.4358	0.5647	0.064*	0.289 (8)
C24A	0.131 (3)	0.3390 (8)	0.4829 (12)	0.052 (6)*	0.289 (8)
H24B	0.1412	0.2829	0.5097	0.063*	0.289 (8)
C25A	0.023 (3)	0.4051 (12)	0.3690 (13)	0.090 (8)*	0.289 (8)
H25B	−0.0477	0.3972	0.3215	0.109*	0.289 (8)
C26A	0.1219 (18)	0.4953 (9)	0.3907 (9)	0.071 (5)*	0.289 (8)
H26B	0.1222	0.5455	0.3550	0.085*	0.289 (8)
C27A	0.2226 (17)	0.5127 (8)	0.4660 (9)	0.062 (5)*	0.289 (8)
C28A	0.3251 (10)	0.6080 (6)	0.4976 (6)	0.052 (3)*	0.289 (8)
H28B	0.3230	0.6601	0.4644	0.063*	0.289 (8)
C29A	0.4198 (12)	0.6278 (7)	0.5680 (6)	0.053 (3)*	0.289 (8)
H29B	0.4205	0.5746	0.6003	0.063*	0.289 (8)
C30A	0.5197 (15)	0.7169 (8)	0.6015 (8)	0.058 (4)*	0.289 (8)
C31A	0.5276 (19)	0.8063 (10)	0.5659 (9)	0.062 (5)*	0.289 (8)
H31B	0.4659	0.8144	0.5182	0.075*	0.289 (8)
C32A	0.697 (5)	0.8440 (13)	0.679 (2)	0.058 (16)*	0.289 (8)
C33A	0.804 (5)	0.8938 (15)	0.743 (2)	0.086 (13)*	0.289 (8)
H33B	0.8472	0.9608	0.7420	0.104*	0.289 (8)
C34A	0.847 (3)	0.8433 (16)	0.8078 (12)	0.104 (10)*	0.289 (8)
H34B	0.9230	0.8760	0.8500	0.125*	0.289 (8)
C35A	0.783 (3)	0.7460 (15)	0.8125 (12)	0.138 (15)*	0.289 (8)
H35B	0.8069	0.7156	0.8607	0.166*	0.289 (8)
C36A	0.678 (2)	0.6898 (10)	0.7431 (10)	0.071 (7)*	0.289 (8)
H36B	0.6433	0.6213	0.7421	0.085*	0.289 (8)
C37A	0.628 (3)	0.7422 (8)	0.6764 (13)	0.052 (6)*	0.289 (8)
C38A	−0.069 (2)	0.2302 (10)	0.3910 (14)	0.103 (8)*	0.289 (8)
H38D	−0.1751	0.2335	0.4048	0.155*	0.289 (8)
H38E	−0.0323	0.1828	0.4235	0.155*	0.289 (8)
H38F	−0.0643	0.2094	0.3291	0.155*	0.289 (8)
N5	0.7895 (4)	0.1618 (3)	0.6196 (2)	0.0802 (9)	
O5	0.7699 (5)	0.1173 (3)	0.6834 (3)	0.1369 (14)	
O6	0.8104 (5)	0.2515 (3)	0.6310 (3)	0.1375 (15)	

### Atomic displacement parameters (Å^2^)

**Table:**

	*U*^11^	*U*^22^	*U*^33^	*U*^12^	*U*^13^	*U*^23^
O4	0.400 (12)	0.290 (8)	0.106 (3)	0.157 (8)	−0.058 (5)	−0.055 (4)
N1	0.0686 (18)	0.0573 (16)	0.0730 (17)	0.0000 (13)	−0.0074 (14)	0.0121 (13)
N2	0.092 (2)	0.0471 (15)	0.0814 (19)	0.0150 (14)	0.0001 (16)	0.0019 (13)
Cl1	0.096 (3)	0.0613 (15)	0.0784 (15)	0.0338 (16)	0.0034 (16)	−0.0078 (11)
S1	0.104 (4)	0.0507 (14)	0.0695 (16)	0.0185 (16)	0.0162 (19)	0.0036 (11)
O1	0.185 (8)	0.086 (4)	0.080 (4)	0.037 (5)	−0.036 (5)	−0.015 (3)
O2	0.114 (5)	0.105 (5)	0.159 (6)	0.048 (4)	0.025 (5)	−0.017 (4)
O3	0.116 (6)	0.058 (4)	0.087 (4)	0.032 (4)	−0.024 (4)	0.010 (3)
C1	0.064 (5)	0.050 (5)	0.049 (5)	0.016 (4)	0.000 (4)	0.007 (3)
C2	0.063 (5)	0.074 (7)	0.076 (8)	0.007 (4)	0.006 (5)	−0.004 (4)
C3	0.094 (8)	0.060 (7)	0.101 (9)	0.011 (5)	−0.003 (6)	0.013 (6)
C4	0.077 (5)	0.045 (5)	0.044 (4)	0.031 (4)	−0.009 (4)	−0.008 (3)
C5	0.066 (6)	0.088 (9)	0.068 (7)	0.029 (5)	−0.006 (5)	0.006 (6)
C6	0.107 (12)	0.104 (11)	0.098 (12)	0.015 (8)	0.002 (8)	0.013 (7)
Cl1A	0.127 (4)	0.0511 (13)	0.136 (3)	0.0177 (18)	0.016 (3)	−0.0226 (14)
S1A	0.099 (3)	0.0487 (13)	0.0677 (18)	0.0154 (15)	0.0049 (16)	0.0110 (11)
O1A	0.155 (6)	0.080 (4)	0.115 (6)	0.026 (4)	0.074 (5)	0.008 (4)
O2A	0.105 (5)	0.076 (4)	0.130 (5)	0.005 (3)	−0.024 (4)	−0.018 (3)
O3A	0.139 (8)	0.071 (4)	0.113 (6)	0.014 (4)	0.045 (5)	0.018 (4)
C1A	0.072 (6)	0.061 (6)	0.056 (5)	0.012 (4)	−0.006 (5)	0.009 (4)
C2A	0.083 (7)	0.078 (7)	0.072 (8)	0.017 (5)	0.013 (5)	−0.004 (4)
C3A	0.082 (7)	0.056 (6)	0.092 (8)	0.001 (5)	0.001 (6)	−0.005 (6)
C4A	0.124 (10)	0.103 (10)	0.097 (10)	0.031 (7)	−0.008 (7)	0.000 (7)
C5A	0.089 (9)	0.081 (9)	0.087 (10)	0.032 (6)	0.009 (7)	−0.013 (5)
C6A	0.050 (5)	0.054 (6)	0.056 (5)	0.008 (4)	0.005 (4)	0.002 (4)
C10	0.077 (2)	0.0585 (19)	0.0656 (19)	0.0147 (16)	0.0016 (17)	0.0026 (15)
C9	0.069 (2)	0.079 (2)	0.068 (2)	0.0069 (18)	0.0054 (16)	0.0175 (18)
C8	0.079 (2)	0.0546 (19)	0.072 (2)	0.0088 (16)	−0.0008 (18)	0.0041 (15)
C7	0.069 (2)	0.0578 (19)	0.072 (2)	0.0114 (15)	0.0063 (16)	0.0085 (15)
C11	0.0589 (18)	0.0594 (18)	0.0585 (17)	0.0113 (14)	−0.0041 (14)	0.0125 (14)
C12	0.065 (2)	0.0570 (18)	0.0650 (18)	0.0143 (15)	0.0060 (15)	0.0106 (14)
C13	0.067 (2)	0.0585 (19)	0.0684 (19)	0.0111 (15)	0.0028 (16)	0.0112 (15)
C14	0.066 (2)	0.0545 (18)	0.0653 (19)	0.0144 (14)	0.0013 (15)	0.0097 (14)
C15	0.090 (3)	0.059 (2)	0.074 (2)	0.0202 (18)	0.0110 (19)	0.0053 (16)
C16	0.0616 (19)	0.0528 (17)	0.073 (2)	0.0091 (14)	−0.0122 (16)	0.0118 (15)
C17	0.067 (2)	0.058 (2)	0.094 (3)	0.0032 (17)	−0.0100 (19)	0.0187 (18)
C18	0.060 (2)	0.084 (3)	0.107 (3)	−0.0039 (19)	−0.005 (2)	0.040 (2)
C19	0.068 (2)	0.086 (3)	0.091 (3)	0.0222 (19)	0.0137 (19)	0.024 (2)
C20	0.062 (2)	0.0618 (19)	0.076 (2)	0.0162 (15)	0.0032 (16)	0.0124 (16)
C21	0.0547 (17)	0.0497 (16)	0.0670 (18)	0.0118 (13)	−0.0075 (14)	0.0104 (13)
C22	0.117 (4)	0.071 (3)	0.111 (3)	−0.025 (2)	−0.003 (3)	0.021 (2)
N3	0.064 (4)	0.093 (6)	0.073 (5)	0.022 (3)	0.006 (2)	−0.007 (3)
N4	0.105 (5)	0.055 (3)	0.092 (4)	0.012 (3)	−0.016 (3)	0.008 (3)
C23	0.081 (4)	0.081 (4)	0.082 (4)	0.013 (4)	0.005 (3)	0.022 (4)
C24	0.070 (6)	0.088 (6)	0.100 (6)	0.014 (3)	0.014 (4)	0.016 (4)
C25	0.078 (5)	0.074 (4)	0.081 (4)	0.025 (4)	0.004 (3)	0.016 (4)
C26	0.084 (5)	0.073 (4)	0.077 (5)	0.019 (3)	0.009 (4)	0.004 (3)
C27	0.062 (3)	0.073 (4)	0.067 (4)	0.019 (3)	0.011 (3)	0.003 (3)
C28	0.084 (4)	0.084 (4)	0.071 (3)	0.027 (3)	0.005 (3)	0.009 (3)
C29	0.086 (4)	0.081 (4)	0.075 (3)	0.024 (3)	0.013 (3)	0.016 (3)
C30	0.070 (3)	0.058 (3)	0.070 (3)	0.018 (3)	0.001 (3)	0.008 (3)
C31	0.097 (5)	0.072 (4)	0.083 (4)	0.019 (4)	−0.006 (3)	0.017 (4)
C32	0.067 (8)	0.070 (8)	0.080 (10)	0.018 (3)	0.011 (3)	0.011 (3)
C33	0.063 (4)	0.066 (5)	0.081 (5)	0.006 (3)	0.002 (4)	0.008 (3)
C34	0.066 (4)	0.068 (4)	0.072 (4)	0.006 (3)	−0.008 (2)	0.011 (3)
C35	0.079 (4)	0.064 (3)	0.065 (3)	0.019 (3)	−0.005 (3)	0.011 (3)
C36	0.064 (4)	0.064 (4)	0.071 (4)	0.010 (2)	0.003 (3)	0.000 (3)
C37	0.062 (4)	0.073 (4)	0.057 (4)	0.023 (3)	0.013 (3)	0.010 (3)
C38	0.073 (4)	0.094 (4)	0.082 (4)	0.012 (3)	−0.008 (3)	−0.015 (4)
N5	0.084 (2)	0.084 (2)	0.075 (2)	0.0217 (18)	−0.0086 (17)	0.0104 (18)
O5	0.143 (3)	0.116 (3)	0.163 (4)	0.028 (2)	0.061 (3)	0.048 (3)
O6	0.129 (3)	0.094 (3)	0.195 (4)	0.014 (2)	−0.024 (3)	0.049 (3)

### Geometric parameters (Å, º)

**Table:**

O4—N5	1.133 (6)	N4—C32	1.372 (6)
N1—C8	1.328 (5)	N4—H1N4	0.8081
N1—C9	1.345 (5)	C23—C24	1.366 (8)
N1—C22	1.476 (5)	C23—C27	1.429 (9)
N2—C15	1.336 (5)	C23—H23A	0.9300
N2—C16	1.377 (5)	C24—H24A	0.9300
N2—H1N2	0.7838	C25—C26	1.376 (11)
Cl1—C4	1.842 (5)	C25—H25A	0.9300
S1—O3	1.342 (4)	C26—C27	1.409 (10)
S1—O1	1.425 (5)	C26—H26A	0.9300
S1—O2	1.427 (7)	C27—C28	1.468 (7)
S1—C1	1.773 (4)	C28—C29	1.318 (6)
C1—C6	1.352 (8)	C28—H28A	0.9300
C1—C2	1.365 (7)	C29—C30	1.404 (7)
C2—C3	1.415 (10)	C29—H29A	0.9300
C2—H2A	0.9300	C30—C31	1.391 (9)
C3—C4	1.343 (9)	C30—C37	1.445 (8)
C3—H3A	0.9300	C31—H31A	0.9300
C4—C5	1.395 (8)	C32—C33	1.377 (6)
C5—C6	1.397 (8)	C32—C37	1.412 (7)
C5—H5A	0.9300	C33—C34	1.362 (8)
C6—H6A	0.9300	C33—H33A	0.9300
Cl1A—C4A	1.842 (5)	C34—C35	1.363 (10)
S1A—O3A	1.342 (4)	C34—H34A	0.9300
S1A—O1A	1.425 (5)	C35—C36	1.443 (9)
S1A—O2A	1.427 (7)	C35—H35A	0.9300
S1A—C1A	1.773 (4)	C36—C37	1.421 (8)
C1A—C6A	1.351 (8)	C36—H36A	0.9300
C1A—C2A	1.365 (7)	C38—H38A	0.9600
C2A—C3A	1.415 (10)	C38—H38B	0.9600
C2A—H2AA	0.9300	C38—H38C	0.9600
C3A—C4A	1.343 (10)	N3A—C25A	1.324 (9)
C3A—H3AA	0.9300	N3A—C24A	1.352 (7)
C4A—C5A	1.395 (8)	N3A—C38A	1.468 (6)
C5A—C6A	1.397 (8)	N4A—C31A	1.326 (8)
C5A—H5AA	0.9300	N4A—C32A	1.372 (6)
C6A—H6AA	0.9300	N4A—H1N4	1.1137
C10—C9	1.356 (5)	N4A—H2N4	0.8600
C10—C11	1.386 (5)	C23A—C24A	1.367 (8)
C10—H10A	0.9300	C23A—C27A	1.429 (9)
C9—H9A	0.9300	C23A—H23B	0.9300
C8—C7	1.360 (5)	C24A—H24B	0.9300
C8—H8A	0.9300	C25A—C26A	1.376 (11)
C7—C11	1.378 (5)	C25A—H25B	0.9300
C7—H7A	0.9300	C26A—C27A	1.410 (10)
C11—C12	1.455 (5)	C26A—H26B	0.9300
C12—C13	1.324 (5)	C27A—C28A	1.468 (7)
C12—H12A	0.9300	C28A—C29A	1.318 (6)
C13—C14	1.432 (5)	C28A—H28B	0.9300
C13—H13A	0.9300	C29A—C30A	1.403 (7)
C14—C15	1.362 (5)	C29A—H29B	0.9300
C14—C21	1.440 (5)	C30A—C31A	1.390 (9)
C15—H15A	0.9300	C30A—C37A	1.445 (8)
C16—C17	1.370 (5)	C31A—H31B	0.9300
C16—C21	1.407 (4)	C32A—C33A	1.377 (6)
C17—C18	1.360 (6)	C32A—C37A	1.412 (7)
C17—H17A	0.9300	C33A—C34A	1.362 (8)
C18—C19	1.404 (6)	C33A—H33B	0.9300
C18—H18A	0.9300	C34A—C35A	1.363 (10)
C19—C20	1.382 (5)	C34A—H34B	0.9300
C19—H19A	0.9300	C35A—C36A	1.443 (10)
C20—C21	1.397 (5)	C35A—H35B	0.9300
C20—H20A	0.9300	C36A—C37A	1.421 (8)
C22—H22A	0.9600	C36A—H36B	0.9300
C22—H22B	0.9600	C38A—H38D	0.9600
C22—H22C	0.9600	C38A—H38E	0.9600
N3—C25	1.324 (9)	C38A—H38F	0.9600
N3—C24	1.352 (7)	N5—O6	1.197 (5)
N3—C38	1.468 (6)	N5—O5	1.216 (5)
N4—C31	1.326 (8)		
			
C8—N1—C9	119.2 (3)	C31—N4—H1N4	118.3
C8—N1—C22	120.5 (3)	C32—N4—H1N4	132.2
C9—N1—C22	120.3 (3)	C24—C23—C27	119.9 (5)
C15—N2—C16	108.9 (3)	C24—C23—H23A	120.0
C15—N2—H1N2	133.0	C27—C23—H23A	120.0
C16—N2—H1N2	117.4	N3—C24—C23	121.6 (6)
O3—S1—O1	128.6 (4)	N3—C24—H24A	119.2
O3—S1—O2	90.3 (5)	C23—C24—H24A	119.2
O1—S1—O2	113.3 (4)	N3—C25—C26	121.2 (5)
O3—S1—C1	110.2 (2)	N3—C25—H25A	119.4
O1—S1—C1	105.7 (2)	C26—C25—H25A	119.4
O2—S1—C1	106.5 (3)	C25—C26—C27	121.2 (5)
C6—C1—C2	120.3 (5)	C25—C26—H26A	119.4
C6—C1—S1	119.1 (4)	C27—C26—H26A	119.4
C2—C1—S1	120.2 (4)	C26—C27—C23	115.5 (4)
C1—C2—C3	119.5 (6)	C26—C27—C28	125.3 (5)
C1—C2—H2A	120.2	C23—C27—C28	119.1 (5)
C3—C2—H2A	120.2	C29—C28—C27	127.1 (4)
C4—C3—C2	119.4 (6)	C29—C28—H28A	116.5
C4—C3—H3A	120.3	C27—C28—H28A	116.5
C2—C3—H3A	120.3	C28—C29—C30	129.7 (4)
C3—C4—C5	119.5 (5)	C28—C29—H29A	115.2
C3—C4—Cl1	121.2 (5)	C30—C29—H29A	115.2
C5—C4—Cl1	119.1 (5)	C31—C30—C29	123.5 (5)
C4—C5—C6	119.8 (5)	C31—C30—C37	104.0 (4)
C4—C5—H5A	120.1	C29—C30—C37	132.4 (5)
C6—C5—H5A	120.1	N4—C31—C30	111.9 (5)
C1—C6—C5	119.9 (5)	N4—C31—H31A	124.1
C1—C6—H6A	120.1	C30—C31—H31A	124.1
C5—C6—H6A	120.1	N4—C32—C33	129.7 (6)
O3A—S1A—O1A	128.5 (4)	N4—C32—C37	107.2 (5)
O3A—S1A—O2A	90.3 (5)	C33—C32—C37	123.1 (6)
O1A—S1A—O2A	113.3 (4)	C34—C33—C32	119.0 (6)
O3A—S1A—C1A	110.2 (2)	C34—C33—H33A	120.5
O1A—S1A—C1A	105.7 (2)	C32—C33—H33A	120.5
O2A—S1A—C1A	106.6 (3)	C33—C34—C35	121.9 (5)
C6A—C1A—C2A	120.4 (5)	C33—C34—H34A	119.1
C6A—C1A—S1A	119.2 (4)	C35—C34—H34A	119.1
C2A—C1A—S1A	120.4 (4)	C34—C35—C36	120.6 (4)
C1A—C2A—C3A	119.5 (6)	C34—C35—H35A	119.7
C1A—C2A—H2AA	120.2	C36—C35—H35A	119.7
C3A—C2A—H2AA	120.2	C37—C36—C35	118.2 (4)
C4A—C3A—C2A	119.4 (6)	C37—C36—H36A	120.9
C4A—C3A—H3AA	120.3	C35—C36—H36A	120.9
C2A—C3A—H3AA	120.3	C32—C37—C36	117.3 (6)
C3A—C4A—C5A	119.5 (5)	C32—C37—C30	107.4 (6)
C3A—C4A—Cl1A	121.3 (5)	C36—C37—C30	135.3 (5)
C5A—C4A—Cl1A	119.2 (5)	C25A—N3A—C24A	120.3 (6)
C4A—C5A—C6A	119.7 (5)	C25A—N3A—C38A	123.0 (7)
C4A—C5A—H5AA	120.1	C24A—N3A—C38A	116.5 (8)
C6A—C5A—H5AA	120.1	C31A—N4A—C32A	109.5 (6)
C1A—C6A—C5A	119.9 (5)	C31A—N4A—H1N4	137.4
C1A—C6A—H6AA	120.1	C32A—N4A—H1N4	112.4
C5A—C6A—H6AA	120.1	C31A—N4A—H2N4	125.3
C9—C10—C11	121.1 (3)	C32A—N4A—H2N4	125.3
C9—C10—H10A	119.4	C24A—C23A—C27A	119.8 (5)
C11—C10—H10A	119.4	C24A—C23A—H23B	120.1
N1—C9—C10	121.0 (3)	C27A—C23A—H23B	120.1
N1—C9—H9A	119.5	N3A—C24A—C23A	121.5 (6)
C10—C9—H9A	119.5	N3A—C24A—H24B	119.2
N1—C8—C7	121.4 (3)	C23A—C24A—H24B	119.2
N1—C8—H8A	119.3	N3A—C25A—C26A	121.1 (6)
C7—C8—H8A	119.3	N3A—C25A—H25B	119.5
C8—C7—C11	121.2 (3)	C26A—C25A—H25B	119.5
C8—C7—H7A	119.4	C25A—C26A—C27A	121.1 (5)
C11—C7—H7A	119.4	C25A—C26A—H26B	119.4
C7—C11—C10	116.0 (3)	C27A—C26A—H26B	119.4
C7—C11—C12	120.1 (3)	C26A—C27A—C23A	115.5 (4)
C10—C11—C12	123.8 (3)	C26A—C27A—C28A	125.2 (5)
C13—C12—C11	125.0 (3)	C23A—C27A—C28A	119.3 (5)
C13—C12—H12A	117.5	C29A—C28A—C27A	127.2 (4)
C11—C12—H12A	117.5	C29A—C28A—H28B	116.4
C12—C13—C14	128.7 (3)	C27A—C28A—H28B	116.4
C12—C13—H13A	115.7	C28A—C29A—C30A	129.8 (4)
C14—C13—H13A	115.7	C28A—C29A—H29B	115.1
C15—C14—C13	122.3 (3)	C30A—C29A—H29B	115.1
C15—C14—C21	105.7 (3)	C31A—C30A—C29A	123.7 (5)
C13—C14—C21	132.0 (3)	C31A—C30A—C37A	104.0 (4)
N2—C15—C14	111.4 (3)	C29A—C30A—C37A	132.2 (5)
N2—C15—H15A	124.3	N4A—C31A—C30A	111.8 (5)
C14—C15—H15A	124.3	N4A—C31A—H31B	124.1
C17—C16—N2	129.1 (3)	C30A—C31A—H31B	124.1
C17—C16—C21	123.4 (4)	N4A—C32A—C33A	129.8 (6)
N2—C16—C21	107.5 (3)	N4A—C32A—C37A	107.2 (5)
C18—C17—C16	117.5 (4)	C33A—C32A—C37A	123.0 (6)
C18—C17—H17A	121.2	C34A—C33A—C32A	119.0 (6)
C16—C17—H17A	121.2	C34A—C33A—H33B	120.5
C17—C18—C19	121.0 (4)	C32A—C33A—H33B	120.5
C17—C18—H18A	119.5	C33A—C34A—C35A	121.8 (5)
C19—C18—H18A	119.5	C33A—C34A—H34B	119.1
C20—C19—C18	121.7 (4)	C35A—C34A—H34B	119.1
C20—C19—H19A	119.2	C34A—C35A—C36A	120.4 (4)
C18—C19—H19A	119.2	C34A—C35A—H35B	119.8
C19—C20—C21	117.9 (3)	C36A—C35A—H35B	119.8
C19—C20—H20A	121.1	C37A—C36A—C35A	118.0 (5)
C21—C20—H20A	121.1	C37A—C36A—H36B	121.0
C20—C21—C16	118.6 (3)	C35A—C36A—H36B	121.0
C20—C21—C14	135.0 (3)	C32A—C37A—C36A	117.3 (6)
C16—C21—C14	106.4 (3)	C32A—C37A—C30A	107.4 (6)
N1—C22—H22A	109.5	C36A—C37A—C30A	135.4 (5)
N1—C22—H22B	109.5	N3A—C38A—H38D	109.5
H22A—C22—H22B	109.5	N3A—C38A—H38E	109.5
N1—C22—H22C	109.5	H38D—C38A—H38E	109.5
H22A—C22—H22C	109.5	N3A—C38A—H38F	109.5
H22B—C22—H22C	109.5	H38D—C38A—H38F	109.5
C25—N3—C24	120.4 (6)	H38E—C38A—H38F	109.5
C25—N3—C38	123.1 (7)	O4—N5—O6	123.5 (6)
C24—N3—C38	116.5 (7)	O4—N5—O5	118.0 (6)
C31—N4—C32	109.5 (6)	O6—N5—O5	118.3 (4)
			
O3—S1—C1—C6	−72.0 (16)	C27—C23—C24—N3	5.6 (17)
O1—S1—C1—C6	70.6 (16)	C24—N3—C25—C26	2.1 (17)
O2—S1—C1—C6	−168.6 (16)	C38—N3—C25—C26	−179.9 (10)
O3—S1—C1—C2	115.4 (12)	N3—C25—C26—C27	0.7 (12)
O1—S1—C1—C2	−102.1 (12)	C25—C26—C27—C23	−0.4 (9)
O2—S1—C1—C2	18.8 (12)	C25—C26—C27—C28	−179.7 (6)
C6—C1—C2—C3	−8 (3)	C24—C23—C27—C26	−2.7 (11)
S1—C1—C2—C3	164.9 (17)	C24—C23—C27—C28	176.7 (9)
C1—C2—C3—C4	15 (3)	C26—C27—C28—C29	0.0 (9)
C2—C3—C4—C5	−13 (3)	C23—C27—C28—C29	−179.3 (6)
C2—C3—C4—Cl1	172.4 (17)	C27—C28—C29—C30	176.8 (6)
C3—C4—C5—C6	3 (3)	C28—C29—C30—C31	−175.9 (7)
Cl1—C4—C5—C6	178 (2)	C28—C29—C30—C37	2.2 (12)
C2—C1—C6—C5	−2 (3)	C32—N4—C31—C30	−0.5 (13)
S1—C1—C6—C5	−174.8 (19)	C29—C30—C31—N4	178.4 (7)
C4—C5—C6—C1	5 (4)	C37—C30—C31—N4	−0.2 (9)
O3A—S1A—C1A—C6A	−60.6 (14)	C31—N4—C32—C33	−178 (2)
O1A—S1A—C1A—C6A	156.9 (13)	C31—N4—C32—C37	0.9 (18)
O2A—S1A—C1A—C6A	36.1 (13)	N4—C32—C33—C34	178.9 (18)
O3A—S1A—C1A—C2A	117.0 (14)	C37—C32—C33—C34	0 (3)
O1A—S1A—C1A—C2A	−25.5 (14)	C32—C33—C34—C35	1 (2)
O2A—S1A—C1A—C2A	−146.3 (14)	C33—C34—C35—C36	−1.1 (13)
C6A—C1A—C2A—C3A	7 (3)	C34—C35—C36—C37	1.3 (9)
S1A—C1A—C2A—C3A	−170.6 (17)	N4—C32—C37—C36	−178.8 (12)
C1A—C2A—C3A—C4A	−15 (3)	C33—C32—C37—C36	1 (3)
C2A—C3A—C4A—C5A	13 (3)	N4—C32—C37—C30	−1.0 (18)
C2A—C3A—C4A—Cl1A	−166.5 (19)	C33—C32—C37—C30	178.4 (19)
C3A—C4A—C5A—C6A	−5 (3)	C35—C36—C37—C32	−1.0 (15)
Cl1A—C4A—C5A—C6A	174.7 (19)	C35—C36—C37—C30	−178.0 (10)
C2A—C1A—C6A—C5A	1 (3)	C31—C30—C37—C32	0.7 (13)
S1A—C1A—C6A—C5A	178.9 (17)	C29—C30—C37—C32	−177.6 (12)
C4A—C5A—C6A—C1A	−2 (3)	C31—C30—C37—C36	177.9 (11)
C8—N1—C9—C10	0.1 (5)	C29—C30—C37—C36	−0.4 (17)
C22—N1—C9—C10	179.7 (4)	C25A—N3A—C24A—C23A	8 (4)
C11—C10—C9—N1	−1.3 (5)	C38A—N3A—C24A—C23A	−177 (3)
C9—N1—C8—C7	0.5 (5)	C27A—C23A—C24A—N3A	−8 (3)
C22—N1—C8—C7	−179.1 (4)	C24A—N3A—C25A—C26A	−1 (5)
N1—C8—C7—C11	0.1 (6)	C38A—N3A—C25A—C26A	−176 (3)
C8—C7—C11—C10	−1.1 (5)	N3A—C25A—C26A—C27A	−5 (4)
C8—C7—C11—C12	179.4 (3)	C25A—C26A—C27A—C23A	4 (3)
C9—C10—C11—C7	1.7 (5)	C25A—C26A—C27A—C28A	−176 (2)
C9—C10—C11—C12	−178.8 (3)	C24A—C23A—C27A—C26A	2 (3)
C7—C11—C12—C13	178.8 (3)	C24A—C23A—C27A—C28A	−177.7 (18)
C10—C11—C12—C13	−0.7 (5)	C26A—C27A—C28A—C29A	179.3 (17)
C11—C12—C13—C14	−178.8 (3)	C23A—C27A—C28A—C29A	−1 (2)
C12—C13—C14—C15	178.9 (3)	C27A—C28A—C29A—C30A	−179.8 (15)
C12—C13—C14—C21	0.0 (6)	C28A—C29A—C30A—C31A	4 (3)
C16—N2—C15—C14	0.3 (4)	C28A—C29A—C30A—C37A	−178 (2)
C13—C14—C15—N2	−179.5 (3)	C32A—N4A—C31A—C30A	−4 (3)
C21—C14—C15—N2	−0.4 (4)	C29A—C30A—C31A—N4A	−178.5 (17)
C15—N2—C16—C17	179.1 (4)	C37A—C30A—C31A—N4A	4 (2)
C15—N2—C16—C21	−0.1 (4)	C31A—N4A—C32A—C33A	−177 (6)
N2—C16—C17—C18	−179.8 (3)	C31A—N4A—C32A—C37A	3 (4)
C21—C16—C17—C18	−0.7 (5)	N4A—C32A—C33A—C34A	179 (5)
C16—C17—C18—C19	−0.2 (6)	C37A—C32A—C33A—C34A	0 (8)
C17—C18—C19—C20	0.9 (6)	C32A—C33A—C34A—C35A	−2 (7)
C18—C19—C20—C21	−0.7 (5)	C33A—C34A—C35A—C36A	7 (4)
C19—C20—C21—C16	−0.2 (5)	C34A—C35A—C36A—C37A	−8 (3)
C19—C20—C21—C14	−179.7 (4)	N4A—C32A—C37A—C36A	179 (3)
C17—C16—C21—C20	0.9 (5)	C33A—C32A—C37A—C36A	−1 (7)
N2—C16—C21—C20	−179.8 (3)	N4A—C32A—C37A—C30A	−1 (4)
C17—C16—C21—C14	−179.4 (3)	C33A—C32A—C37A—C30A	179 (5)
N2—C16—C21—C14	−0.1 (3)	C35A—C36A—C37A—C32A	5 (4)
C15—C14—C21—C20	179.9 (4)	C35A—C36A—C37A—C30A	−175 (3)
C13—C14—C21—C20	−1.1 (6)	C31A—C30A—C37A—C32A	−2 (3)
C15—C14—C21—C16	0.3 (4)	C29A—C30A—C37A—C32A	−179 (3)
C13—C14—C21—C16	179.3 (3)	C31A—C30A—C37A—C36A	179 (3)
C25—N3—C24—C23	−5 (2)	C29A—C30A—C37A—C36A	1 (5)
C38—N3—C24—C23	176.6 (12)		

### Hydrogen-bond geometry (Å, º)

*Cg*3, *Cg*6, *Cg*7 and *Cg*9 are the centroids of the C16–C21, C32–C37, N4*A*/C30*A*–C32*A*/C37*A* and C32*A*–C37*A* rings, respectively.

**Table:**

*D*—H···*A*	*D*—H	H···*A*	*D*···*A*	*D*—H···*A*
N2—H1*N*2···O3*A*	0.78	2.19	2.937 (9)	161
N4—H1*N*4···O4^i^	0.81	2.43	3.220 (11)	165
N4—H1*N*4···O5^i^	0.81	2.32	2.987 (8)	141
C3*A*—H3*AA*···O5^ii^	0.93	2.43	3.246 (13)	146
C8—H8*A*···O2*A*^iii^	0.93	2.40	3.213 (8)	146
C10—H10*A*···O5^iv^	0.93	2.51	3.234 (6)	134
C18—H18*A*···O1*A*^v^	0.93	2.52	3.345 (8)	148
C22—H22*A*···O1*A*^iii^	0.96	2.45	3.368 (9)	160
C22—H22*C*···O2*A*^vi^	0.96	2.32	3.082 (9)	136
C26—H26*A*···O6^vii^	0.93	2.53	3.440 (7)	168
C15—H15*A*···*Cg*6^vii^	0.93	2.71	3.550 (6)	151
C15—H15*A*···*Cg*7^vii^	0.93	2.94	3.844 (10)	165
C15—H15*A*···*Cg*9^vii^	0.93	2.83	3.656 (13)	149
C34—H34*A*···*Cg*3^ii^	0.93	2.78	3.602 (7)	149
C38—H38*C*···*Cg*6^ii^	0.96	2.95	3.714 (8)	137
C38—H38*C*···*Cg*9^vii^	0.96	2.83	3.627 (14)	141
C34*A*—H34*B*···*Cg*3^ii^	0.93	2.89	3.56 (2)	130

Symmetry codes: (i) *x*, *y*+1, *z*; (ii) −*x*+2, −*y*+1, −*z*+1; (iii) −*x*+1, −*y*, −*z*; (iv) −*x*+1, −*y*, −*z*+1; (v) −*x*+2, −*y*+1, −*z*; (vi) *x*, *y*−1, *z*; (vii) −*x*+1, −*y*+1, −*z*+1.
